# The benefits of impossible tests: Assessing the role of error-correction in the pretesting effect

**DOI:** 10.3758/s13421-021-01218-6

**Published:** 2021-08-06

**Authors:** Tina Seabrooke, Chris J. Mitchell, Andy J. Wills, Angus B. Inkster, Timothy J. Hollins

**Affiliations:** 1 Department of Psychology, University of Southampton, Hampshire, Southampton, SO17 1PS UK; 2 Department of Psychology, University of Plymouth, Plymouth, UK

**Keywords:** Tests, Errors, Generation, Learning, Memory

## Abstract

Relative to studying alone, guessing the meanings of unknown words can improve later recognition of their meanings, even if those guesses were incorrect – the pretesting effect (PTE). The error-correction hypothesis suggests that incorrect guesses produce error signals that promote memory for the meanings when they are revealed. The current research sought to test the error-correction explanation of the PTE. In three experiments, participants studied unfamiliar Finnish-English word pairs by either studying each complete pair or by guessing the English translation before its presentation. In the latter case, the participants also guessed which of two categories the word belonged to. Hence, guesses from the correct category were semantically closer to the true translation than guesses from the incorrect category. In Experiment 1, guessing increased subsequent recognition of the English translations, especially for translations that were presented on trials in which the participants’ guesses were from the correct category. Experiment 2 replicated these target recognition effects while also demonstrating that they do not extend to associative recognition performance. Experiment 3 again replicated the target recognition pattern, while also examining participants’ metacognitive recognition judgments. Participants correctly judged that their memory would be better after small than after large errors, but incorrectly believed that making any errors would be detrimental, relative to study-only. Overall, the data are inconsistent with the error-correction hypothesis; small, within-category errors produced better recognition than large, cross-category errors. Alternative theories, based on elaborative encoding and motivated learning, are considered.

## Introduction

Tests are frequently administered by educators as a means of both formative and summative assessment. With a summative assessment, the primary aim is usually to assess how much knowledge a student has retained at the end of a semester or module (Dixson & Worrell, 2016). With a formative assessment, by contrast, a stronger emphasis is placed on the opportunity to *learn* from any mistakes that were made during that test. Thanks to over a century of basic learning and memory research, we now know that taking an initial formative test often improves performance on a later, summative test – a pattern that is known as the “testing effect” (for reviews, see Dunlosky et al., 2013; Roediger & Butler, 2011). These studies demonstrate that tests are potent learning tools, and many researchers now strongly encourage the use of tests in educational settings (e.g., Agarwal et al., 2014; McDaniel et al., 2007; Roediger III et al., 2011).

A question that has received some recent interest is whether retrieval must be successful to produce a testing effect, or whether any retrieval attempt will enhance subsequent learning, relative to study alone. To date, most studies that have explored the effects of unsuccessful retrieval attempts on learning have used a procedure developed by Kornell et al. (2009). In this procedure, participants first attempt to remember weakly associated word pairs such as *whale-mammal* and *tide-beach*. On Read-only trials, the participants simply study the pair for the full trial duration. On Test trials, the participants are first shown the cue (e.g., *whale*) and are then asked to guess the target (e.g., *dolphin*) before the correct target (*mammal*) is revealed. In a subsequent cued-recall test, participants usually recall more targets from the Test condition than the Read-only condition – guessing improves memory. Importantly, this pattern is observed even when only the incorrectly guessed targets from the Test trials are included in the analysis (Kornell et al., 2009). Kornell et al.'s (2009) procedure was designed to emulate a scenario in which a student generates an incorrect answer to a question that relates to a familiar concept. This scenario has been termed *“unsuccessful retrieval”* and has been widely researched in recent years (e.g., Carneiro et al., 2018; Cyr & Anderson, 2015; Hays et al., 2013; Kornell, 2014; Richland et al., 2009; Vaughn et al., 2017; Vaughn & Rawson, 2012; for a review, see Kornell & Vaughn, 2016).

A further set of studies have shown that even guessing the meaning of *completely novel* cue words can improve memory. In Potts and Shanks' (2014) experiments, for example, participants attempted to learn the common English definitions of rare English words (e.g., *roke-mist*) or vocabulary from an unfamiliar foreign language such as Euskara (e.g., *gatza-cheese*). Similar to Kornell et al.'s (2009) procedure, on Test trials, participants were presented with a cue (e.g., *gatza*) and had to guess the target definition (*cheese*). Guessing the definition of a cue word before reading the true definition improved performance on a subsequent target multiple-choice test (relative to just studying the definitions). The cues were novel when presented at encoding, and so participants’ responses were likely to be “pure” guesses, rather than informed predictions (see Brod, 2021).

A range of terms has been used to refer to Potts and Shanks' (2014) guessing effect (e.g., “errorful generation” – Potts & Shanks, 2014; “test-potentiated learning” – Hays et al., 2013). In the current article, we use the term *pretesting effect* (PTE; see, e.g., Richland et al., 2009). We acknowledge that Richland et al.'s (2009) PTE study was observed with text-based materials, and participants were required to learn facts rather than vocabulary. However, we prefer the term *pretesting effect* over *errorful generation* and *test-potentiated learning* because it is somewhat more accessible, and goes some way to providing a simple and intuitive description of the procedure.

The current work focuses on the role of *error-correction* in the vocabulary learning PTE task described by Potts and Shanks (2014). Error-correction is thought by theorists within both the learning and memory literatures to play a major role at encoding (e.g., Brod et al., 2018; Carrier & Pashler, 1992; De Loof et al., 2018; Fazio & Marsh, 2009; Metcalfe, 2017; Rescorla & Wagner, 1972; Wagner, 1981). According to the error-correction idea, the learning system is engaged when there is a discrepancy between an (incorrect) prediction and the actual target that is presented (e.g., Wagner, 1981). This same idea has been applied in the memory literature, where incorrect guesses appear to enhance the processing of immediate corrective feedback (Grimaldi & Karpicke, 2012). One important prediction that can be derived from the error-correction theory is that learning will be proportionate to the size of the error – the error magnitude. That is, guesses that are semantically far away from the target will generate a larger error signal than errors that are semantically close to the target, and will therefore result in better learning. Below, we present existing data suggesting that the unsuccessful retrieval effect (Kornell et al., 2009) – with familiar cues and targets – is not driven by an error-correction mechanism. We then present three new experiments that sought to assess the role of error-correction in the PTE, using novel cues (Potts & Shanks, 2014; Richland et al., 2009; Seabrooke, Hollins, et al., 2019a).

It is important to note that the error-correction hypothesis under scrutiny here is silent with respect to phenomenology and metacognitive processes. Participants may be aware that the learning system has been triggered by an error signal and are perhaps surprised to find that their prediction is wrong, but this is not necessary to the model. Rather, error-correction is simply an algorithm to describe when learning does, and does not, take place – based on the objective discrepancy (the semantic distance) between the guess and the target. One way to envisage this algorithm working, in terms of cognitive processes, is that the guess increases attention to, and processing of, the target when it is revealed because the participant is surprised (see e.g., Potts et al., 2019; Seabrooke, Mitchell, et al., 2019b; Zawadzka & Hanczakowski, 2019). According to this interpretation, the more surprised the participant is, the more target processing will occur. However, this description is just one way to view the error correction process – it is not intrinsic to the model.

We also recognize here that there is an important distinction between *objective* and *subjective* error magnitude, the latter of which may be closely related to contextual factors such as surprise and confidence. In the pretesting paradigm, participants may not have much confidence in their guesses, and therefore may not be surprised to learn that their guesses were wrong. This low level of confidence and surprise may reduce the likelihood of an error-correction mechanism being triggered, especially when compared to other paradigms in which the participants generate informed predictions (see Brod, 2021). Indeed, the participants may even be more surprised if they generate a guess that is *close* to the true answer in the pretesting paradigm (i.e., the perception of a *near miss*). For the present purposes, we characterize the error correction account from an objective error magnitude standpoint that is based on the semantic distance between the guess and the target (see also Grimaldi & Karpicke, 2012). In the *General discussion*, however, we provide a broader discussion of objective versus subjective (or perceived) error magnitude.

Previous studies that used Kornell et al.'s (2009) unsuccessful retrieval paradigm compared the learning of semantically related (e.g., *whale-mammal*) and unrelated (e.g., *pond-spanner*) word pairs (Grimaldi & Karpicke, 2012; Huelser & Metcalfe, 2012; Knight et al., 2012). Intuitively, participants should generate larger errors (i.e., guesses that are semantically further away from the target) when guessing the targets from unrelated word pairs than related word pairs. According to the error-correction hypothesis, then, guessing should confer the largest benefit on unrelated pairs, where the semantic distance between the guess and target is greatest. The typical finding, however, is quite different; unsuccessful retrieval attempts typically only improve subsequent cued recall of targets from semantically *related* word pairs (Grimaldi & Karpicke, 2012; Huelser & Metcalfe, 2012; Knight et al., 2012). This finding, that the guessing benefit is seen for related but not unrelated cue-target word pairs, is a key line of support for a quite different account of unsuccessful retrieval: *search set theory* (Grimaldi & Karpicke, 2012)*.* Search set theory suggests that, when a cue such as “*whale*” is presented on a Test trial, it will bring to mind many associated words, such as “*ocean,*” “*mammal,*” “*large,*” and “*dolphin.*” Although a participant might incorrectly guess “*dolphin*” on that trial, the correct target (“*mammal*”) will nevertheless have received activation as part of the participant’s “search set” of related concepts. This activation of the true target “*mammal*” during the guessing stage may then result in better encoding of that target when it is later presented. Of course, when the cue and target are unrelated (e.g., *whale-bicycle*), the search set is very unlikely to include the target (*bicycle*), and so no memory benefit will be observed. The absence of a guessing effect for unrelated materials is, therefore, consistent with search set theory and not an error-correction learning mechanism.

Zawadzka and Hanczakowski (2019) provided further evidence for search set theory (and against an error-correction mechanism) in their first two experiments. They used homograph cues that could be interpreted correctly or incorrectly. For example, the cue “*arms*” could be paired with either the target “*legs*” or “*missile.*” If a participant guessed “*hands,*” the interpretation would be correct if the target was “*legs,*” but incorrect if the target was “*missile.*” In a subsequent cued-recall test, participants only showed a benefit of guessing when the cue was interpreted correctly (i.e., when the guess was related to the target and so the semantic distance between the guess and the target was comparatively low). No benefit was seen when the interpretation was wrong and the guess was unrelated to the target. Similar to Grimaldi and Karpicke's (2012) finding described above, then, guessing was only beneficial for related cues and targets (for related research, see Cyr & Anderson, 2018).

An exception to the pattern described above comes from a recent study by Metcalfe and Huelser (2020). They observed a beneficial effect of guesses even when the cue and target were unrelated. The cues were word pairs that included a homograph and a second word that disambiguated the homograph. Hence, using the homograph *palm*, the cue word pair *wrist-palm* (but not *tree-palm*) would be congruent with the target *hand*. Conversely, *tree-palm* would be congruent with the target *coconut* (but not *hand*). On a cued-recall test (both cue words were presented – e.g., *wrist-palm*), participants who generated a (wrong) guess about the target at encoding showed a benefit not only for congruent materials (in which the cue and target were related), but also for the incongruent materials where the cue and target were unrelated (e.g., the cue *wrist-palm* paired with the target *tree*). Hence, under certain conditions, cue-target relatedness is not crucial to the demonstration of an unsuccessful retrieval effect (although we note that at least one of the cues was always related to the targets in these experiments). Importantly for the current discussion, however, the effect seen on incongruent (i.e., large error) trials was no different from that seen on congruent trials; there was no benefit gained from guesses associated with a larger (semantically distant) error.

Although there is little evidence for an error-correction mechanism in Kornell et al.'s (2009) unsuccessful retrieval effect, there are two related reasons to suppose that this result may not generalize to the PTE: the familiarity of the cues and the use of a cued-recall test. The problem with familiar cues is that they will be associated with many related concepts (and particularly the participant’s guess), which may oppose any effects of error-correction. If the cue *pond* is presented at encoding, for example, the participant may guess *lily*. In a subsequent cued-recall test, *pond* is likely to activate *lily* once again. If the target is unrelated to the cue (e.g., the target is *pond-spanner*), then retrieval of the guess at test may create interference and oppose any benefit from the larger error magnitude experienced at encoding. The use of novel cues, as in Potts and Shanks’ (2014) study, may resolve this problem; the cue will not be so strongly associated with the guess, and so the guess will be less likely to interfere with memory for the target on test. Hence, using novel cues in the PTE paradigm may reveal evidence for an error-correction mechanism.

Another important feature of Kornell et al.'s (2009) procedure is the use of the *cued-recall* test. While larger guessing errors may indeed hinder performance on cued-recall tests (e.g., by providing a relatively weak mediator between the cue and target), the learning mechanisms that are activated by these large errors may nevertheless facilitate the encoding of the *target* in memory. In a target recognition test, by contrast, such interference would not be expected to play such a large role, and so a benefit of larger errors on target encoding may now be revealed.

There is some evidence to support the idea that error magnitude might have different effects, depending on whether the final test assesses cue-target associative memory (e.g., cued-recall) or simple target memory. While Zawadzka and Hanczakowski (2019) found benefits of generating small, semantically related errors over large, unrelated errors when the final test was a cued-recall test (Experiments 1 and 2 – see above), a different pattern was observed in independent cue tests (Experiments 3 and 4). These independent cues were semantically related to the original cue and target, but were not presented at encoding. Since the independent cues were not presented at encoding, the recall test assessed *target* memory rather than memory for the original cue-target associations. Under these circumstances, participants showed a benefit of guessing both when their interpretation of the cue was correct and when it was incorrect. That is, guessing boosted target memory regardless of the size of the error. However, most importantly from the current perspective, the guessing benefit was no greater in the large-error condition (incorrect interpretation of the homograph cue) than it was in the small-error condition (correct interpretation of the cue). Hence, there was no evidence that large magnitude errors generated better memory performance than small magnitude errors.

In sum, the data reviewed above suggest that error-correction mechanisms play no role in Kornell et al.’s (2009) unsuccessful retrieval effect. Previous work almost always asked participants to study familiar cues, with the final criterion test almost always cued recall. As we have noted above, these factors may not be best suited to uncover evidence of an error-correction mechanism. What we aim to test here is whether error-correction plays a role when the cues are novel (e.g., foreign words), and therefore participants tend to generate pure guesses rather than informed predictions, as in Potts and Shanks' (2014) pretesting procedure.

In the present work, we assessed target recognition (a non-associative measure) for two reasons. Firstly, target recognition memory is less likely to suffer from interference from guesses that are unrelated to the target. Secondly, past studies of the PTE using unfamiliar cue-target word pairs have shown that the effect is only observed in tests that assess target memory (Seabrooke et al., 2021; Seabrooke, Hollins, et al., 2019a).

## Experiment 1

The present experiments tested the error-correction hypothesis in the PTE using Finnish words (for which the participants should have no strong associates). Each experiment followed the basic format of an encoding phase, followed by a test phase. During the encoding phase, participants were asked to learn the English translations of Finnish words. To manipulate error magnitude, the targets were selected from two semantic categories: four-footed animals and items of clothing. On Pretest trials, participants were presented with a Finnish word and were asked to guess the semantic category that the word belonged to (four-footed animal or item of clothing), before guessing the English translation and receiving corrective feedback. Hence, although the target guess would usually be wrong, participants could guess the correct category (a *within-category error*) or the incorrect category (a *cross-category error*). On cross-category error trials, there should be greater semantic distance between the guess and the target than on within-category error trials. Hence, according to the error-correction mechanism of learning, cross-category errors should produce better encoding of the target. Lastly, on Read-only trials, the participants simply studied the Finnish word and its English translation for the full trial duration. Participants then completed an old-new target recognition test, where the targets from the encoding phase were mixed with novel foils, and participants had to determine whether each word was new or old.

### Method

#### Participants

A sample of size of 72 participants was chosen before data collection. In our previous experiments on the PTE in target recognition, our average observed effect size was Cohen’s *d*_*z*_ = 0.61 (Seabrooke, Hollins, et al., 2019a). We did not have a clear *a priori* estimate of the effect size for the difference in recognition between targets from within- and cross-category error trials, but we did anticipate that any such effect would be smaller than the overall difference in recognition of targets from Pretest (collapsed across within- and cross-category error trials) and Read-only trials. As a conservative estimate, we therefore selected our sample size to detect a within- versus cross-category error effect size that was half the effect size that was seen for Pretest versus Read-only targets in Seabrooke, Hollins, et al. (2019a). The chosen sample size of 72 participants provides good power (> 80%) to obtain an effect size of Cohen’s *d*_*z*_ = 0.305. One participant was replaced because they did not make enough within- and cross-category errors (see below). The final sample consisted of 60 females and 12 males, who were recruited from the University of Plymouth and were aged between 18 and 50 years (*M* = 21.13 years, *SD* = 6.88 years). The participants were psychology undergraduates who completed the study for partial course credit. The pool of undergraduate participants typically contains many more females than males, which explains the skew towards females in our experiments.

#### Apparatus

The experiment was programmed in E-Prime 2.0 (https://pstnet.com/) and was presented on a 22-in. computer monitor. Stimuli were presented on a white background, and responses were made using a standard keyboard. The participants wore headphones throughout the experiment (to muffle any external noise).

#### Stimuli

The word pairs consisted of 36 four-footed animals and 36 items of clothing. To maximize the number of errors that participants would generate on Pretest encoding trials, we selected targets that did not appear in the list of exemplars that were identified as being frequently generated in Van Overschelde et al.'s (2004) category norms. We also selected targets that did not bear resemblance to the Finnish translation. Each word pair was randomly allocated to the Pretest, Read-only or foil condition for each participant.

#### Procedure

Before the encoding phase, participants were told that their task was to learn the English translations of Finnish words. The participants were first presented with eight practice trials (four Pretest trials and four Read-only trials, using two animal and two clothing targets within each encoding condition) in a random order. They then completed the main encoding phase, which consisted of 40 Pretest and 12 Read-only trials, which were randomly intermixed. We included more Pretest trials than Read-only trials to increase the likelihood that participants would make enough cross- and within-category errors for a meaningful analysis of the final test data. Within each encoding condition (Pretest/Read-only), half of the targets were four-footed animals, and the remainder were items of clothing.

**Fig. 1:**
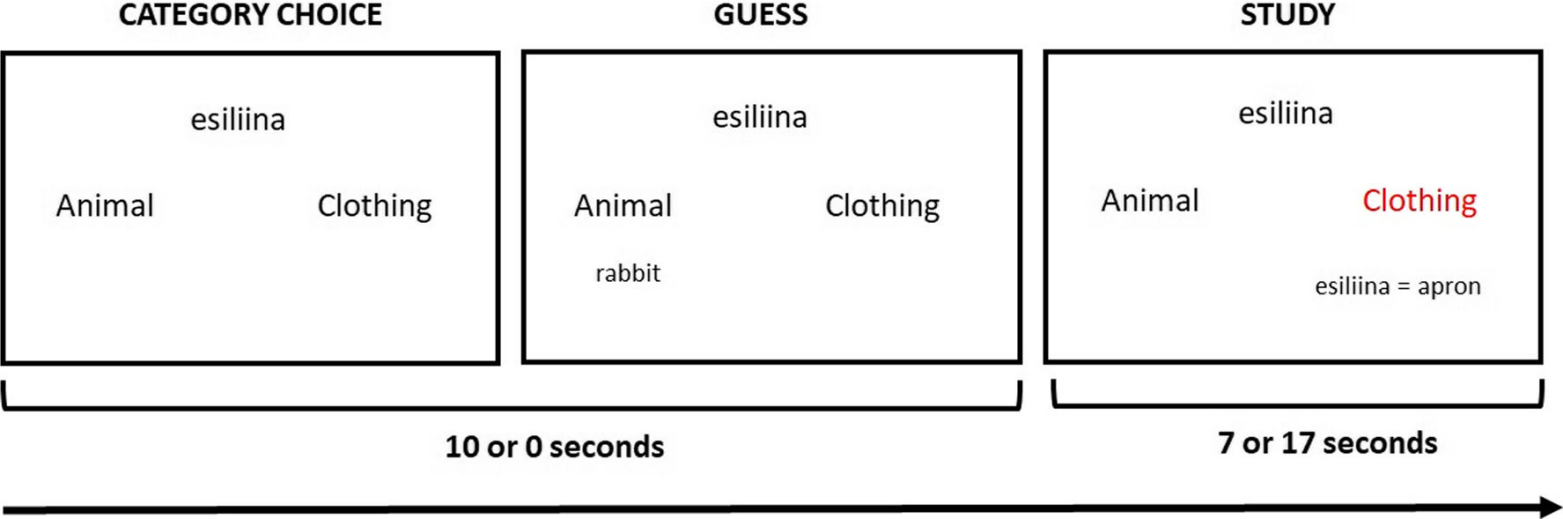
Schematic representation of encoding phase trials (Experiment 1)**.** *Note.* On Pretest trials, participants were presented with a cue (e.g., *esiliina*) and had 10 s to guess the category (animal or clothing) and at least the first three letters of the target. The participants’ guesses appeared on screen as they typed. After 10 s, the cue and the target (e.g., *esiliina = apron*) were presented together for a further 7 s. On Read-only trials, the category choice and guess phase were omitted and the cue and the target were presented together in the study phase for 17 s

Figure 1 depicts an example trial from the encoding phase. On Pretest trials, a cue (a Finnish word, e.g., *esiliina*) was first presented at the top center of the screen. The two categories (“Animal” and “Clothing”) were also presented on the left- and right-center of the screen. The participants first guessed the category that the cue belonged to by pressing the left or right arrow key on the computer keyboard. After selecting a category, the question “Which [four-footed animal/article of clothing] do you think this is?” appeared beneath the chosen category. The participants had to guess the target (the English translation) by typing either a four-footed animal or an item of clothing. These guesses appeared on the screen as they typed, beneath the question. The participants had a total of 10 s to guess the category and the English translation. The participants were able to press the Backspace key to change their answer until the 10 s had elapsed. Before the encoding phase, the experimenter strongly encouraged the participants to type at least the first three letters of their guess (although they could type more if they wished). After 10 s, the question and guess were replaced by the cue and the correct target (e.g., *esiliina = apron*) for 7 s. The feedback was presented beneath the correct category, which was presented in red. If the participant did not choose a category or type at least three letters of their guess on the Pretest trials, they received a warning message after the feedback. The Read-only trials, which were presented for 17 s (to match the total trial duration of the Pretest trials), included just the feedback of the Pretest trials (i.e., the complete word pair presented beneath the correct category, which was highlighted in red – see Fig. 1). Trials were separated by intervals that varied randomly between three and four seconds.

All targets from the Read-only condition were presented again in the subsequent target recognition test. The targets from Pretest trials were only allocated to the target recognition test if the participant had selected a category (animal or clothing) and submitted at least a three-letter guess that did not match the first three letters of the target. These measures were adopted to ensure that the participants committed an error on each Pretest trial that was allocated to the target recognition test. The experiment aborted after the encoding phase if the participant failed to generate at least 12 within- and cross-category errors each (this happened for one participant). If more than 12 within- or cross-category errors were generated, a random 12 targets from each error type were selected for presentation at test. The remaining 12 items that were not presented at encoding (six animal targets and six clothing targets) were presented as foils during the test. Thus, the target recognition test consisted of 12 foils, 12 targets from Read-only trials, and 24 targets from Pretest trials (12 trials from cross- and within-category error trials each). The test trials were randomly intermixed. The experimenter verbally explained the test instructions to the participants, but the test phase otherwise took place immediately after the encoding phase (i.e., the retention interval averaged a few minutes).

On each trial during the target recognition test, a target (e.g., *apron*) was presented at the top-center of the screen, above the question, “Did you see this word before?” Yes/No options were presented beneath the question, and the participants responded by clicking on an option with the mouse. Responding was not time-limited. The target recognition test was preceded by eight practice trials, using targets from the practice encoding trials. The cues and targets were presented in size 16 Verdana font and in lowercase throughout each experiment in this paper.

### Results

On average during the encoding phase, the participants generated within-category errors on 47.67% (*SD* = 6.83%), and cross-category errors on 45.28% (*SD* = 7.55%), of Pretest trials. On the remaining Pretest trials, the participants either failed to generate at least a three-letter guess or guessed at least the first three letters of the correct target. The targets from these trials were not presented during the target recognition test.

**Fig. 2:**
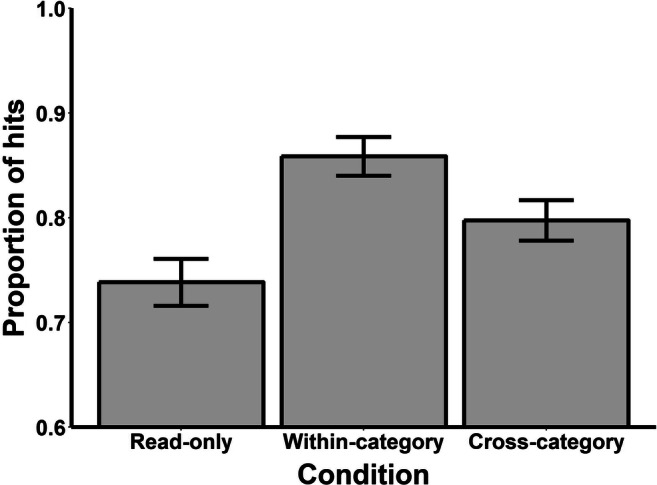
Mean proportion of hits to old targets during the target recognition test of Experiment 1. *Note*. Error bars represent difference-adjusted, within-subject 95% confidence intervals (Baguley, 2012)

Figure 2 shows the mean proportion of hits to targets from Read-only, within-category and cross-category error trials in the target recognition test. Since the foils were novel words that were not presented at encoding (i.e., they were not related to any encoding condition), any differences between conditions in discrimination (*d’*) and response bias (*c*) scores must reflect differences in the hit rates. We therefore took the average proportion of false alarms, and the proportion of hits from each encoding condition, as our measures of interest. The average proportion of false alarms was 0.07 (*SD* = 0.10), suggesting that the participants were very good at recognizing that the foils were novel. A one-way ANOVA on the proportion of hits to old targets revealed an overall effect of trial type, *F* (2, 142) = 17.77, mean square error (*MSE*) = 0.01, *p* < .001, generalized eta square (ɳ_g_^2^) = .08. Pairwise comparisons revealed that the targets from both within-category, *t* (71) = 5.74, *p* < .001, *d*_*z*_ = 0.68, and cross-category, *t* (71) = 2.71, *p* = .008, *d*_*z*_ = 0.32, error trials were recognized more often than targets from Read-only trials. Furthermore, the participants correctly recognized more targets from within-category error trials than cross-category error trials, *t* (71) = 3.49, *p* < .001, *d*_*z*_ = 0.41.

### Discussion

Overall, incorrectly guessing the English translations of Finnish words produced better subsequent recognition of those English translations than studying them without first guessing. This pattern was seen regardless of whether the errors were close or far away from the correct translation. This finding is consistent with the first prediction of the error-correction hypothesis. Contrary to the second prediction of the error-correction hypothesis, however, larger errors did not improve target recognition compared to smaller errors. In fact, the opposite pattern was observed; recognition memory was best following a within-category (smaller) error.

The observation that errors improve subsequent target recognition, even for cross-category errors, mirrors the pattern that is seen for unrelated and novel word pairs such *pond-spanner* and *roke-mist* (Potts et al., 2019;Seabrooke et al., 2021 ; Seabrooke, Hollins, et al., 2019a). We have previously argued that this result is also inconsistent with search set theory (Seabrooke et al., 2021; Seabrooke, Hollins, et al., 2019a). Search set theory suggests that unsuccessful guessing attempts activate both the participant’s overt guess and other related concepts, including the correct target. This target is therefore processed more effectively when it is revealed than targets that were presented on Read-only trials (where the target was not partially activated during a guessing attempt). Importantly, the target should only be activated by the search set mechanism when the cue and target are related, because it is only under these circumstances that the search set should include the target. Thus, search set theory predicts that pretests should only improve memory for semantically related word pairs such as *pond-frog.* Although search set theory was designed to explain cued recall effects, in which this pattern is observed, the theory predicts the same result for target recognition tests. This is because the locus of the effect is on the *target*; incorrect guesses activate the target (when the cue and target are related), which improves processing of that target (e.g., Grimaldi & Karpicke, 2012). Improved target processing on Pretest trials (relative to Read-only trials) should improve both cued recall *and* target recognition, but only for targets that were paired with semantically related cues. The fact that generating erroneous guesses improves subsequent target recognition when guessing the meaning of unfamiliar Finnish words therefore provides evidence against search set theory.

Overall, the current results contradict the predictions of both the search set hypothesis and the error-correction hypothesis. Before considering other explanations, we first sought to establish that the effects were robust. To this end, in Experiment 2 we attempted to first replicate the target recognition results of Experiment 1. We also tested whether the results would generalize to an associative recognition test. As discussed above, previous research has demonstrated that, for semantically unrelated word pairs (from the participants’ perspective), pretesting does not improve performance on associative tests of memory such as cued recall or associative recognition (Seabrooke et al., 2021; Seabrooke, Hollins, et al., 2019a). It remains possible, however, that the benefit of within- over cross-category errors reflects a different psychological process to the process that is responsible for the general benefit that is seen for generating errors over studying. Perhaps a close guess would provide additional intrinsic motivation to study the translation closely when it is revealed, thereby improving subsequent associative recognition of word pairs from within-category errors compared to cross-category errors. This intrinsic motivation account, which we discuss further in the *General discussion*, could also explain the benefit of within- over cross-category errors that was observed in the target recognition test of Experiment 1 (increased processing of the target after a close guess would be expected to improve target recognition as well). In terms of associative recognition, within-category guesses may also serve as more effective mediators than cross-category guesses, thereby allowing participants to recall the cue-target associations more successfully on within-category error trials than cross-category error trials. Finally, it is also possible that cue-target associations will be more easily encoded following within-category errors than cross-category errors, because participants need only process the target (not the category as well) after a within-category error. If any of these possibilities are correct, an associative recognition test may usefully dissociate the general effect of making an error from whatever process differentially affects learning following large and small errors.

Moreover, Experiment 1 demonstrated that, relative to the Read-only condition, within-category errors were more beneficial for target recognition than cross-category errors. In Seabrooke, Hollins, et al. (2019a) experiments, all errors at encoding were likely to be cross-category errors, because participants were not provided with the target category when they were asked to guess the definition of a rare English word. Since the within-category (vs. Read-only) effect appears to be larger than the comparable cross-category effect, an associative effect of pretesting may be easier to detect for within-category errors than cross-category errors. We administered an associative recognition test rather than a cued-recall test because we were concerned that the participants’ guesses (within- or cross-category errors) would produce different degrees of interference in a cued-recall test.

## Experiment 2

Participants in Experiment 2 completed the same encoding phase as in Experiment 1. Half of the participants then completed an old-new target recognition test, as in Experiment 1. The remaining participants completed an associative recognition test, which we have used in previous work (Seabrooke, Hollins, et al., 2019a). Here, participants were presented with Finnish-English word pairs that were either presented intact (i.e., as they were studied at encoding) or re-arranged (i.e., a Finnish word from the encoding phase was presented with a different target from the encoding phase). In this task, associative memory is required to distinguish intact word pairs from re-arranged pairs, but any interference from the participants’ guesses during the encoding phase should be minimal (because participants are not required to actively retrieve the target at test). This procedure also has the advantage of reducing the likelihood of a floor effect at test (cued recall performance is often very poor in these experiments – see, e.g., Seabrooke, Hollins, et al., 2019a), which would restrict our ability to observe any effect of error magnitude.

### Method

#### Participants, apparatus, and materials

A sample size of 44 participants per group was determined before data collection. This sample size has good power to detect the effect size of the within- versus cross-category effect seen in Experiment 1 (85% power at *d*_*z*_ = 0.41). Thus, 88 psychology undergraduates from the University of Plymouth took part in the experiment for course credit. Six participants failed to generate enough within and cross-category errors to progress onto the test phase, and another withdrew from the experiment because of illness. These participants were replaced. The final sample consisted of 44 participants per group. There were 71 females and 17 males, who were aged between 18 and 52 years (*M* = 21.08 years, *SD* = 6.44 years). The apparatus and stimuli were as in Experiment 1.

#### Procedure

The procedure for the target recognition group was the same as that used in Experiment 1, except that all trials were separated by fixed 1,500-ms intervals. The encoding phase for the associative recognition group was identical to the encoding phase for the target recognition group. For the associative recognition test, six word pairs from each error type were randomly chosen and were allocated to a “paired” list. These items retained their original pairing when they were presented at test. The remaining word pairs were allocated to the “re-paired” list. The targets from these word pairs were swapped with targets from another (randomly chosen) word pair from the re-paired list. Table 1 shows some example trials. The re-paired cue and target were always from the same error type (within/cross category), but they were randomly selected from either category (animal or clothing). Similarly, six randomly selected word pairs from the Read-only condition were allocated to the paired list and were presented intact during the test phase. The remaining six word pairs from the Read-only condition were allocated to the re-paired list, and the targets from these word pairs were swapped in the same way as for the re-paired word pairs from the within- and cross-category error conditions.

**Table 1:** Example associative recognition trials in Experiment 2

Trial type	Encoding		Test	
	Paired	To be re-paired	Paired	Re-paired
Read-only	poro - reindeer tossut - slippers	huntu - veil mursu - walrus	poro - reindeer tossut - slippers	huntu - walrus mursu - veil
Within-category	smokki - tuxedo esiliina - apron	mäyrä - badger kaapu - robe	smokki - tuxedo esiliina - apron	mäyrä - robe kaapu - badger
Cross-category	apina - monkey balettihame - tutu	kruunu - crown sadetakki - raincoat	apina - monkey balettihame - tutu	kruunu - raincoat sadetakki - crown

Each associative recognition test trial began with the presentation of a word pair (e.g., *esiliina = apron*), the statement “Were these words presented together?” and “yes” and “no” options. The word pair was presented in the top-center of the screen, the question was presented centrally, and the response options were presented in the bottom center of the screen. Participants had to select a response option using the mouse (responding was not time-limited). The test phase began with eight practice trials (four paired and four re-paired trials), using the cues and targets from the practice encoding trials. The participants were told whether their answers were correct or not on the practice trials to emphasize that the task was to determine whether the cues and targets had been presented *together* at encoding, not simply whether they had been presented at all. The main associative recognition test consisted of 36 trials, comprising 12 word pairs each from the within-category error condition, cross-category error condition, and Read-only condition (half of which came from the paired list, the remainder of which came from the re-paired list). The trials were randomly intermixed and were separated by 1500ms intervals. No feedback was provided during the main test.

### Results

On average during the encoding phase, the participants generated within-category errors on 46.11% (*SD* = 7.85%), and cross-category errors on 46.88% (*SD* = 6.90%), of Pretest trials. As in Experiment 1, the participants failed to generate a suitable error on the remaining Pretest trials. The targets from these trials were not allocated to either test.

**Fig. 3:**
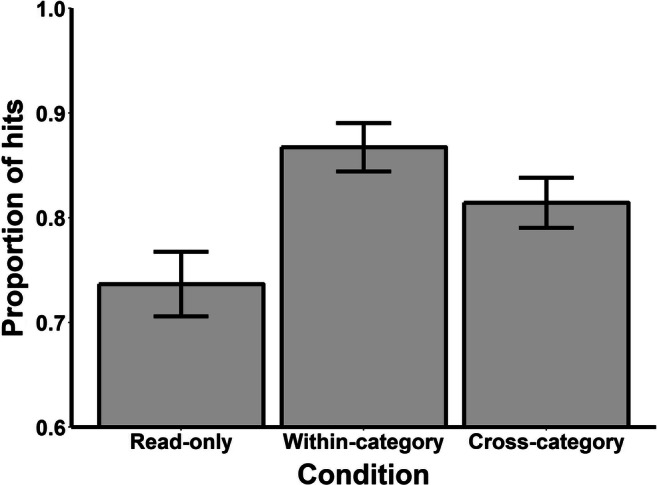
Mean proportion of hits to old targets during the target recognition test of Experiment 2. *Note.* Error bars represent difference-adjusted, within-subject 95% confidence intervals (Baguley, 2012)

The analysis strategy from Experiment 1 was adopted for the target recognition test. The mean proportion of false alarms was 0.07 (*SD* = 0.10), suggesting that the participants were good at identifying the foils as novel. Figure 3 shows the mean proportion of hits per trial type. A one-way ANOVA on the proportion of hits to old targets revealed an overall effect of trial type, *F* (2, 86) = 12.80, *MSE* = 0.01, *p* < .001, ɳ_g_^2^ = 0.11. Pairwise comparisons revealed that participants correctly recognized more targets from both within-category, *t* (43) = 4.68, *p* < .001, *d*_*z*_ = 0.70, and cross-category, *t* (43) = 2.70, *p* = .01, *d*_*z*_ = 0.41, error trials than Read-only trials. Furthermore, the participants correctly recognized more targets from within-category error trials than cross-category error trials, *t* (43) = 2.59, *p* = .01, *d*_*z*_ = 0.39. Thus, the target recognition data replicate those of Experiment 1.

In the associative recognition test, the foils (re-paired word pairs) were unique to each trial type (Read-only trials, within-category error trials, and cross-category error trials). Discrimination (*d’*) and response bias (*c*) scores were therefore taken as the primary measures. Table 2 shows the mean hit rate, false alarm rate, *d’*, and *c* scores in the associative recognition test. No significant effects of trial type were observed for either *d’*, *F* (2, 86) = 1.39, *MSE* = 0.57, *p* = .25, ɳ_g_^2^ = .02, or *c*, *F* (2, 86) = 1.99, *MSE* = 0.16, *p* = .14, ɳ_g_^2^ = .03. Bayesian ANOVA, using the R package *BayesFactor* (Morey & Rouder, 2018) indicated substantial evidence for the null (BF < 1/3) in the case of *d’*, BF_10_ = 0.24, with an inconclusive result (1/3 < BF < 3) in the case of *c*, BF_10_ = 0.52. For a direct comparison of the cross- and within-category conditions, there was Bayesian evidence for the null, both for *d’*, BF = 0.22, and for *c*, BF = 0.23. The remaining pairwise Bayesian comparisons were inconclusive.

**Table 2:** Mean HR, FAR, d’ and c rates in the associative recognition test of Experiment 2

	Read-only	Within-category	Cross-category
HR	0.73 (0.69, 0.76)	0.74 (0.71, 0.78)	0.74 (0.70, 0.78)
FAR	0.31 (0.27, 0.35)	0.40 (0.36, 0.45)	0.40 (0.36, 0.44)
*d’*	1.26 (1.08, 1.43)	1.02 (0.85, 1.18)	1.03 (0.88, 1.18)
*c*	-0.07 (-0.15, 0.01)	-0.23 (-0.31, -0.14)	-0.21 (-0.30, -0.12)

*HR* hit rate, *FAR* false alarm rate, *d’* discrimination, *c* response bias. Numbers in parentheses denote difference-adjusted, within-subject, 95% confidence intervals (Baguley, 2012)

### Discussion

Experiment 2 fully replicated the target recognition results of Experiment 1. Participants recognized more targets for which they had generated both within- and cross-category errors than those that they had simply studied. Targets from within-category error trials were also recognized more often than targets from cross-category error trials. This second finding is again inconsistent with the error-correction hypothesis. For the associative recognition test, by contrast, no significant effects of encoding condition were observed, with Bayesian evidence for the null in the case of *d’*. This result suggests that, relative to an equivalent period of study time, pretesting has no impact on the quality of cue-target associative learning. Further analysis indicated that the differential effect of error magnitude observed for target recognition does not extend to associative recognition (with Bayesian evidence for the null for both *d’* and *c* in this case). Thus, Experiment 2 provides no evidence to suggest that the mechanism that produces the overall PTE is different to the one that produces the differential effect that is seen for within- and cross-category errors.

It might seem odd that we observed a marked effect of error magnitude on target memory, but not on associative memory. The two might seem to be intrinsically linked. In fact, in one dominant model of associative memory (Wagner, 1981), associative strength is a product of the extent to which the target is processed. We return to the issue of associative versus target strength in the *General discussion*. Before that, we report an attempt to ascertain the extent to which participants can judge their learning across the three trial types.

## Experiment 3

In Experiment 3, we sought to examine whether participants were aware of the benefits of generating within-category errors over both cross-category errors and just studying. Several previous studies have shown that participants often do not appreciate the benefits of generating errors during learning (Huelser & Metcalfe, 2012; Potts & Shanks, 2014; Yang et al., 2017; Zawadzka & Hanczakowski, 2019). Potts and Shanks' (2014) participants, for example, consistently gave lower judgments of learning to pretested word pairs than to pairs that were studied alone. This pattern was observed even though pretesting consistently *improved* target memory. A recent survey of North American undergraduates further suggests that students often do not engage in pretesting in genuine pedagogical environments (Pan et al., 2020). Interestingly, 91% of students felt that it was either moderately or very important to avoid generating errors when studying. When practice questions were made available, just 14% of students said that they attempted those questions before studying, as opposed to 74% of students stating that they attempted the questions *after* studying the topic. In contrast, 96% of students agreed that studying feedback after making errors was either moderately or very helpful. Thus, while students often avoid making errors, they do appreciate the educational value of learning from errors.

Experiment 3 aimed to test whether participants’ beliefs about the effects of generating large and small errors on target recognition would match their target recognition performance. One reason why participants may undervalue pretests is because their metacognitive judgments may be based on ease of processing or *processing fluency* (Potts & Shanks, 2014). After generating a cross-category error, participants must process both the category that the target belonged to *and* the target itself. Following a within-category error, by contrast, participants need only process the target itself. Thus, there is less information to process on within-category error trials than cross-category error trials. With this in mind, we predicted that participants would give higher metacognitive memory judgments for targets that were presented after within-category errors than targets that were presented after cross-category errors. Consistent with previous work (e.g., Huelser & Metcalfe, 2012; Yang et al., 2017), we predicted that participants would give the highest metacognitive judgments on Read-only trials, since these trials require the least processing. In sum, we predicted that participants’ judgments would be based on fluency of the information presented on each trial. They would therefore be incorrect with respect to the benefits of guessing in general (Pretest trials vs. Read-only trials), but correct with respect to the benefits of a close guess (within-category) over a distant guess (cross-category).

### Method

The method was the same as the method for the target recognition group in Experiment 2, except in the following respects.

#### Participants

A sample size of 46 participants was determined before data collection. This sample size has good power to detect a within- versus cross-category error effect of the average effect sizes seen in the target recognition tests of Experiments 1 and 2 (85% power at *d*_*z*_ = 0.40). Thus, 46 participants were recruited from the University of Plymouth for either course credit or £4 each. Three participants were replaced because they failed to generate enough within- and cross-category errors. The final sample consisted of 39 females and seven males, who were aged between 18 and 51 years (*M* = 20.83 years, *SD* = 5.16 years).

#### Procedure

The participants completed the same encoding phase as in Experiment 2, but they also made trial-by-trial recognition predictions after studying each word pair. Specifically, the participants answered the question, “How confident are you that you will recognize that English word definition when it is presented later?” by typing a number between zero (*No chance I’ll recognize it*) and 100 (*I’ll definitely recognize it*). The target recognition test was the same as in Experiment 2.

### Results

On average during the encoding phase, the participants generated within-category errors on 45.65% (*SD* = 7.75%), and cross-category errors on 45.11% (*SD* = 7.45%), of Pretest trials. As in the earlier experiments, the participants failed to generate a clear within- or cross-category error on the remaining Pretest trials, and the targets from these trials were not presented at test.

**Fig. 4:**
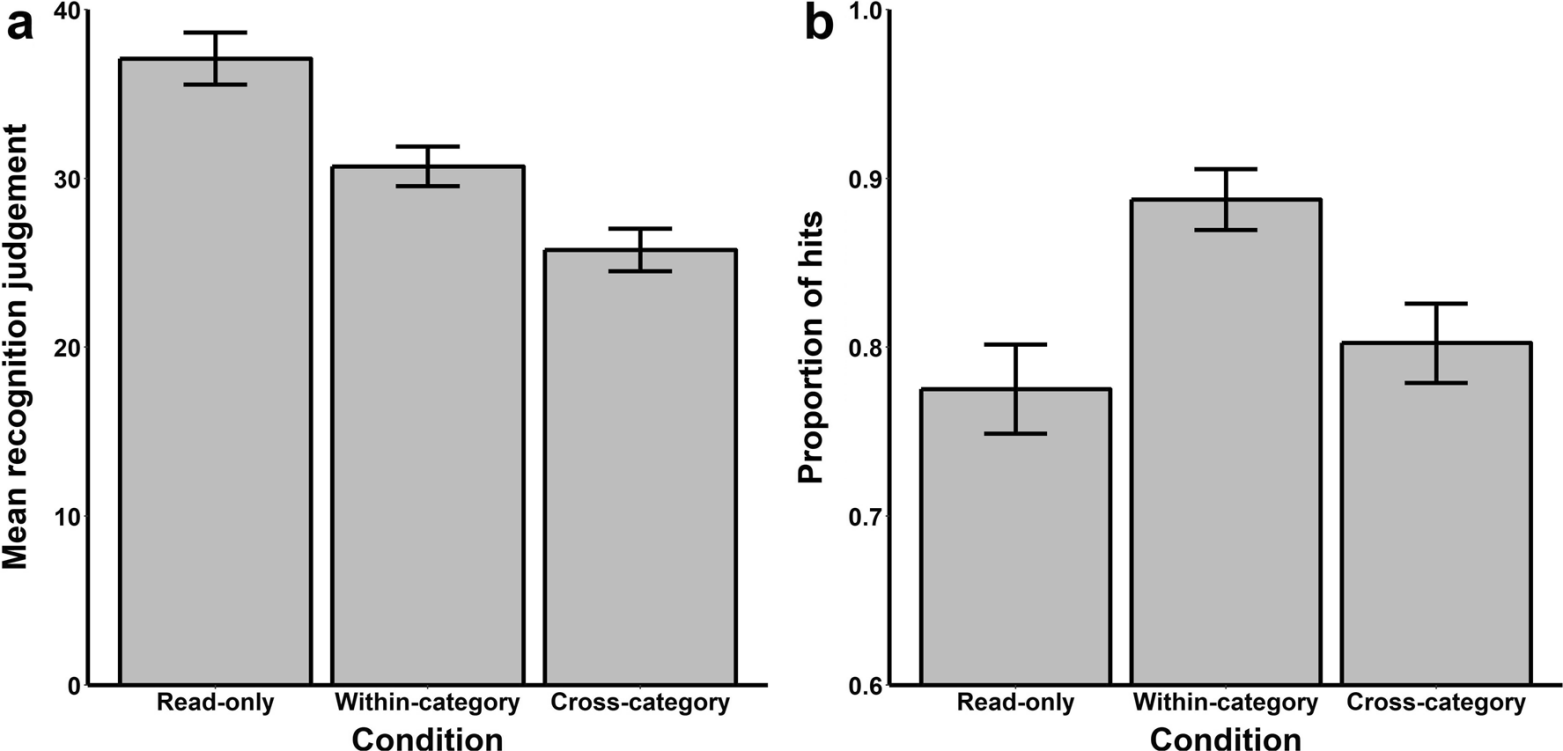
Results of Experiment 3. *Note.* Panel A depicts the mean recognition judgments for targets during the encoding phase. Ratings of zero and 100 represent “No chance I’ll recognize it” and “I’ll definitely recognize it,” respectively. Panel B depicts the mean proportion of hits to old targets during the target recognition test. Error bars represent difference-adjusted, within-subject 95% confidence intervals (Baguley, 2012)

Figure 4a depicts the mean recognition predictions from Read-only trials and all Pretest trials in which the participants generated within- and cross-category errors at encoding. A one-way ANOVA revealed an overall effect of trial type, *F* (2, 90) = 36.64, *MSE* = 40.50, *p* < .001, ɳ_g_^2^ = .11. Follow-up *t*-tests revealed that participants gave significantly higher recognition predictions for targets from Read-only trials than targets from within-category error trials, *t* (45) = 4.56, *p* < .001, *d*_*z*_ = 0.67, and cross-category error trials, *t* (45) = 7.70, *p* < .001, *d*_*z*_ = 1.13. Furthermore, the participants gave significantly higher recognition predictions for targets from within-category error trials than targets from cross-category error trials, *t* (45) = 4.60, *p* < .001, *d*_*z*_ = 0.68.

In the target recognition test, participants were generally good at recognizing that the foils were novel; the average proportion of false alarms was 0.05 (*SD* = 0.09). Figure 4b shows the mean proportion of hits to old targets from Read-only, within-category and cross-category error trials in the target recognition test. There was an overall effect of trial type, *F* (2, 90) = 13.37, *MSE* = 0.01, *p* < .001, ɳ_g_^2^ = .12. Pairwise comparisons revealed that participants recognized more targets from within-category error trials than Read-only trials, *t* (45) = 5.07, *p* < .001, *d*_*z*_ = 0.75. The difference in recognition of targets from cross-category error trials was not significantly different from recognition of targets from Read-only trials, although the pattern was numerically in the same direction as in Experiments 1 and 2, *t* (45) = 1.02, *p* = .31, *d*_*z*_ = 0.15. Finally, as in Experiments 1 and 2, participants correctly recognized more targets from within-category error trials than cross-category error trials, *t* (45) = 4.58, *p* < .001, *d*_*z*_ = 0.68.

### Discussion

Consistent with Experiments 1 and 2, targets that were presented on within-category error trials at encoding were more likely to be recognized in a subsequent target recognition test than targets that were presented on either Read-only or cross-category error trials. Similar to Experiments 1 and 2, participants also showed a tendency to recognize more targets from cross-category errors trials than Read-only trials, although this pattern did not reach statistical significance in Experiment 3. One possibility is that the recognition predictions at encoding somehow affected participants’ behavior on Read-only and/or cross-category error trials. Participants might, for instance, have encoded Read-only targets more effectively after having made a metacognitive judgment, thereby producing a reduced recognition difference between Read-only and cross-category error trials (see also Soderstrom et al., 2015). In general, we cannot rule out the possibility that the participants’ recognition predictions affected their studying behavior.

As we predicted, the recognition predictions were only partially in line with participants’ performance in the target recognition test. First, the participants correctly gave higher recognition predictions to targets from within-category error trials than cross-category error trials. This pattern is consistent with their performance on the target recognition test and suggests that they were aware of the benefits of close errors on subsequent recognition. The highest predictions, however, were given to the Read-only targets, which were recognized significantly *less* well than the targets from the within-category error condition. This latter result is consistent with previous studies, in which judgments of learning were higher for word pairs that were merely read than for word pairs for which participants generated errors at encoding (Huelser & Metcalfe, 2012; Potts & Shanks, 2014; Yang et al., 2017; Zawadzka & Hanczakowski, 2019). Together, these studies show that there is a mismatch between participants’ performance on memory tests and their beliefs about the most effective studying techniques.

## General discussion

Three experiments examined the role of error magnitude in a novel modification of a pretesting task. In each experiment, participants were given the task of learning the English translations of Finnish words. Each translation was from one of two categories: four-footed animals or items of clothing. When learning the word pairs, participants either studied the word pair for the full trial duration (Read-only condition), or guessed the category and the translation before the correct translation was revealed (Pretest condition). Since the participants were not previously exposed to the word pairs, their guesses were usually wrong on Pretest trials. When examining the types of errors made, participants’ guesses could be classified as either large errors (when they guessed an exemplar from the wrong category) or small errors (when they guessed an incorrect exemplar from the correct category). Across all three experiments, incorrect guesses produced better subsequent recognition performance than study alone. This result replicates previous demonstrations of the benefits of pretesting (e.g., Potts & Shanks, 2014; Seabrooke, Hollins, et al., 2019a). The more important observation, from the perspective of testing the error-correction account of the PTE, was that (small) within-category errors produced better subsequent target recognition than (larger) cross-category errors. This result provides clear evidence against the error-correction hypothesis.

The effects described above were observed using target recognition as the dependent variable. In contrast, no effects of pretesting were observed in an associative recognition task in Experiment 2. It might be argued that there is something peculiar to our novel encoding procedure – where participants had to guess the category of the target as well as the target itself on Pretest trials – that might have generated the very different pattern of results on the target recognition and associative recognition tasks. However, previous experiments, using more standard encoding procedures, have shown very similar results (Seabrooke et al., 2021; Seabrooke, Hollins, et al., 2019a). In these earlier experiments, participants were not required to guess the category of the target on Pretest trials, but simply to guess the target – the standard approach used by Potts and Shanks (2014). Again, guessing benefitted target recognition but not cue-target associative memory for word pairs involving unfamiliar cues. Across paradigms, then, the consensus is that pretesting improves target familiarity, but not associative memory, of unrelated word pairs. We provide one possible reason for this pattern of results at the end of the *General discussion*.

Finally, participants were asked to make recognition predictions during the encoding phase in Experiment 3. They tended to predict (incorrectly) that targets presented on Read-only trials would produce better memory than targets that were presented on Pretest trials. This finding is consistent with previous observations that participants are unaware of the benefits of guessing, relative to study alone (Huelser & Metcalfe, 2012; Potts & Shanks, 2014; Yang et al., 2017; Zawadzka & Hanczakowski, 2019). Participants were correct, however, in predicting that targets presented on within-category error trials would be better remembered than those presented on cross-category error trials. These additional results have important implications for the analysis presented below of the main findings.

Our main aim was to test the idea at the heart of the error-correction hypothesis - that larger errors committed during a generation attempt would increase subsequent processing of the correct answer (e.g., Wagner, 1981). This account makes a clear prediction that feedback that follows a large error should be better remembered than feedback that follows a small error (because it will be processed more effectively). Our data show the opposite result, and therefore speak directly against this prediction.

As discussed in the *Introduction*, several previous studies have already provided evidence to suggest that lower magnitude errors benefit memory more than high magnitude errors. Studies using the original unsuccessful retrieval paradigm (Kornell et al., 2009), for example, showed reliable effects of guessing on subsequent cued recall of related, but not unrelated, word pairs (Grimaldi & Karpicke, 2012; Huelser & Metcalfe, 2012; Knight et al., 2012). In this procedure, participants’ guesses for related items are more likely to have low error magnitude than those for unrelated items; given the cue *pond*, it would be easier to make a guess that is close to the target *frog* (in the related case) than to the target *spanner* (in the unrelated case). This finding – that guessing boosts memory for related word pairs (where the error is likely to be small) but not unrelated word pairs (where the error will be larger) – is clearly inconsistent with the error-correction approach. A similar pattern was reported in Zawadzka and Hanczakowski’s (2019) first two experiments. Here, guessing improved subsequent cued recall when the guess related to the “correct” interpretation of a homograph cue, but not when the “incorrect” interpretation was assumed. Again, this suggests that, when the guess is close to the true answer, a larger memory benefit is observed. Overall, then, the error-correction hypothesis receives little support from prior research on unsuccessful retrieval in cued recall, or from the current experiments investigating the effects of pretesting on recognition memory.

Our results are, however, inconsistent with the results of Zawadzka and Hanczakowski’s (2019) Experiments 3 and 4. In those experiments, the participants completed the same encoding phase as in their first two experiments (making congruent and incongruent errors) but, in the final test, the participants had to recall the target in response to an independent cue that was semantically related to both the original cue and the target. Thus, the test assessed memory for the targets rather than the original cue-target associations. Given the nature of the final test and our findings, it might be expected that congruent errors would produce better subsequent target memory than incongruent errors. By contrast, the authors found that congruent and incongruent errors improved target memory equally, relative to Read-only trials. Unlike in our experiments, then, small (congruent) errors did not produce better subsequent target memory than large (incongruent) errors.

Zawadzka and Hanczakowski (2019) used a quite different procedure to the procedure used in our experiments, and there are at least two major procedural differences that could explain the discrepant results. First, Zawadzka and Hanczakowski presented participants with familiar cues that had two interpretations (e.g., “arms”), while we presented participants with unfamiliar cues for which the participants should have had no strong associates (e.g., “esiliina”). Second, the final test formats differed substantially, with Zawadzka and Hanczakowski employing an independent cue test, while we employed a more straightforward target recognition test. We cannot say at this stage which of those factors are important for observing larger memory effects for small errors than large errors. However, the relative importance of these factors could be tested by incorporating Zawadzka and Hanczakowski’s materials in our experiment (e.g., present “arms” at encoding and ask participants to guess whether the target relates to a body part or a weapon, followed by a target recognition test). Conversely, our foreign vocabularly materials could be followed by Zawadzka and Hanczakowski’s independent cue test. These would both be interesting avenues for further research. Although we observed slightly different results from Zawadzka and Hanczakowski’s Experiments 3 and 4, the takeaway message from these experiments is that large errors do not seem to boost learning, over and above small errors. If anything, targets presented after small errors improve target memory more so than large errors.

We should note at this point that the research presented here focused on the pretesting paradigm, where participants generated guesses to questions about which they had no relevant information. Thus, the participants’ guesses were likely to be “pure” and possibly random guesses, and the participants probably did not have much confidence in those guesses. The evidence for the error-correction hypothesis in this pretesting paradigm is not compelling. However, it has been argued that having confidence in one’s response is necessary to observe surprise-based prediction errors and the subsequent beneficial effects on learning (Brod, 2021). Thus, the error-correction theory may well find support in paradigms that require participants to make informed predictions (e.g., answering familiar trivia questions) rather than guesses. Our data do not speak to the psychological mechanisms that underpin the effects found in such paradigms.

### Theoretical accounts of the pretesting effect (PTE)

We believe that there are at least two theories that can account for the pattern of data observed in our experiments. The first explanation is based on the *elaborative generation hypothesis* (Potts et al., 2019). According to this view, pretesting activates other concepts, which then become associated with both the cue and the correct target when it is revealed. Importantly, and unlike other popular theories such as *search-set theory* (Cyr & Anderson, 2018; Grimaldi & Karpicke, 2012; Hays et al., 2013; Kornell et al., 2009; Zawadzka & Hanczakowski, 2019), the elaborative generation hypothesis does not necessitate that the cue and the target be *semantically* related. So long as the cue brings other related concepts to mind (as evidenced by the participants’ guesses on Pretest trials), these concepts should become associated with the target. Thus, the elaborative generation hypothesis suggests that pretesting improves memory for targets (relative to an equivalent study period) because additional representations become activated during the generation attempt, which then become associated with both the cue and the target (Potts et al., 2019).

In our target recognition tests, of course, the targets were presented without the cues, and hence there was little opportunity for such a cue-mediator-target chain to operate (Potts et al., 2019; Seabrooke, Hollins, et al., 2019a; Seabrooke, Mitchell, et al., 2019b). Perhaps the chain could operate in a backward fashion, with the presentation of old targets activating associated mediators (guesses) from the encoding phase, and thereby allowing the target to be correctly categorized as “old.” Targets from Read-only trials would not be expected to benefit from mediation of this kind, because no mediators were established on Read-only trials at encoding. Moreover, the participants’ guesses should have been semantically closer to the targets on within-category error trials than cross-category error trials. Stronger guess-target associations may well form for guesses that are closely related to the target, leading to better recognition of targets from within-category error trials than cross-category error trials (as was observed). In other words, while semantic relatedness does not appear to be crucial to a observe a PTE (at least in target recognition tests), the size of the effect may still be modulated by the semantic relationship between the guess and the target.

The elaborative generation hypothesis still, however, predicts that pretesting should improve memory for the associations between cues and targets. Indeed, the elaborative generation account is intrinsically associative in nature. However, no associative recognition effect was observed in Experiment 2, and this pattern is consistent with past failures to see associative effects of pretesting in both associative recognition and cued recall tasks (Seabrooke, Hollins, et al., 2019a). Thus, the elaborative generation hypothesis goes only so far in providing a general account of the PTE.

A second explanation of the PTE is that participants are more motivated to pay attention to targets that are revealed on Pretest trials (Potts et al., 2019; Seabrooke, Mitchell, et al., 2019b). Pretesting might, for example, increase motivation to learn by providing a “metacognitive reality check” that highlights the fact that the participant does not know the answer once they are required to generate a response (Carpenter & Toftness, 2017). The recognition predictions that were recorded in Experiment 3 provide some insight into this possibility. Here, participants predicted that their recognition of Read-only targets would be better than that of Pretest targets. This intuition could have reduced the extent to which they attended to (or rehearsed/processed) Read-only targets, and hence reduced recognition performance at test. However, the main novel finding from our study does not fit well with this proposal. Participants also predicted that they would recognize within-category error targets better than cross-category error targets – and they were correct in this judgment. If the extent to which participants attend to the target is driven by a metacognitive reality check, then targets from the cross-category condition (where larger errors were made) should have been attended to, and therefore recognized, especially well. Alternatively, one might argue that any reality check would occur during the guessing attempt (i.e., before the presentation of corrective feedback), and therefore the nature of the target (whether it is in the same or a different category from the guess) should be irrelevant. Either way, while the metacognitive reality check hypothesis can explain why targets from Pretest trials are recognized more often than targets from Read-only trials, it struggles to explain the added advantage of generating within-category errors over cross-category errors.

A related possibility is that participants believe that, although it is very unlikely, they may have guessed correctly on Pretest trials. They would then be more interested (motivated and curious) to find out what the true target was on these trials. An increase in curiosity, motivation, and perhaps even low-level excitement at the possibility of being correct may be enough to increase processing at encoding and hence target recognition at test. This possibility is consistent with the finding that participants rate their curiosity (Potts et al., 2019) and motivation (Seabrooke, Mitchell, et al., 2019b) to discover targets that they have guessed more highly than targets that they have not guessed. It is also consistent with Gruber et al.'s (2014) finding that participants show better memory for information that they are more curious about. This “motivational” account of the PTE can readily explain why targets from Pretest trials (collapsed across error type) were better recognized than targets from Read-only trials in the present experiments; participants paid more attention to the targets because they were more motivated to study them.

To explain why within-category errors increased recognition even more than cross-category errors, the motivational account described above would have to further suppose that generating a “close” error is especially motivating. In fact, there is evidence from gambling research that near misses (close guesses) are particularly potent triggers of emotion and motivation (Sharman & Clark, 2016; Wadhwa & Kim, 2015). Unlike in gambling scenarios, correct guesses in our experiments were not associated with any extrinsic payoff such as money. Nevertheless, a close guess on a very difficult task (e.g., guessing the meaning of an unfamiliar foreign word) may provide enough *intrinsic* reward to produce an (perhaps low-level) emotional and motivational response. In this way, target processing and later recognition may be enhanced. Overall, then, this motivational account captures the two main current findings: better memory for targets presented on Pretest than Read-only trials, and better memory after within-category errors than cross-category errors. It remains to be seen why this enhanced target encoding effect does not translate into an associative memory benefit (e.g., in Experiment 2 here), but, as noted above, this is a difficulty that applies equally to all current accounts of the PTE. Below, we present one possible reason for why pretesting may improve target but not associative memory for unrelated materials.

### Target versus associative memory

Experiment 2 of the current series adds to a growing consensus that pretesting improves item (cue and target) memory, but not associative memory, for semantically unrelated materials (Seabrooke et al., 2021; Seabrooke, Hollins, et al., 2019a). The one exception to this narrative is that pretesting appears to improve memory for more complex, unrelated materials such as trivia questions (Kornell, 2014) and essays on unfamiliar topics (Richland et al., 2009). As noted above, existing theories of the PTE struggle to explain these findings. We do not have a conclusive explanation at present, but one possibility is that the benefit of pretesting on memory for unrelated materials is small, and item memory tests are simply more sensitive than associative memory tests. The PTE for more complex materials may be larger (and therefore easier to detect with associative memory tests) than the effect seen for simple word pairs, particularly if the PTE is driven by motivation, curiosity, or interest. It seems reasonable to anticipate that participants would be more interested to learn the answers to trivia questions than simple word pairs. In sum, associative memory tests may simply not be sensitive enough to detect the benefits of pretesting that are seen with item memory tests.

### Conclusion

The current data add to previous findings showing that pretesting with novel cues can improve target recognition. Most importantly, the data contradict popular error-correction accounts of the PTE. When participants made a guess that was close to the target (a small, within-category error), their subsequent target recognition was better than when the guess was further away from the target (a large, cross-category error). Two accounts of these effects were proposed. The elaborative generation hypothesis suggests that participants’ guesses become linked in memory to the target, thereby providing additional evidence during the recognition test to suggest that the target is an old item. The attentional, or motivational, account instead suggests that participants are curious to know whether their guesses are correct, which focuses attention to the target, thereby enhancing subsequent recognition of those targets. This performance benefit is amplified in cases where their guess is close to the true answer. While both accounts explain most of the current data, neither satisfactorily explains why the PTE is not observed in associative memory tests (for semantically unrelated materials). Our data suggest that pretesting will not help students to learn simple and novel associations, although pretesting may well help students to learn more complex and educationally relevant information (Richland et al., 2009). Understanding why pretesting does not improve associative learning for simple materials is an important outstanding question for the future.
